# 

**Published:** 1874-03-15

**Authors:** 


### BOOKS RECEIVED,

*Through Jansen, McClurg & Co., Chicago.*

The Puerperal Diseases. By Fordyce Barker. 576 pp. New York: D. Appleton & Co.

Handbook of Medical and Surgical Reference. Wythe. New York: Wm. Wood & Co.

Wythe's Pocket Dose Book. Philadelphia: Lindsay & Blackiston.

An Introduction to Physical Measurements, etc. By Dr. Kohlrousch. Translated from the German. New York: D. Appleton & Co.

Sanitary Associations During the Franco-German War — 1870–71. By Thos. W. Evans, M.D., etc.

Dictionary of Elevations, and Climatic Register of the United States. By J. M. Toner, M.D. New York: Van Nostrand, 23 Murray and 27 Warren sts. Price, in paper, $3.00; cloth, $3.75.

*From W. B. Keen, Cooke & Co., Chicago.*

Lectures on the Chemical Uses of Electricity. By J. Russell Reynolds, M.D., etc. Philadelphia: Lindsay & Blakiston.

Transaction of Wisconsin State Medical Society, 1873.

Transactions of Indiana State Medical Society, 1873.

